# SAMS-Net: A Smoothness-Anchored Monotone Neural Differential Equation Network for Failure-Only-Supervised Structural Health Indicator Construction

**DOI:** 10.3390/s26123640

**Published:** 2026-06-07

**Authors:** Yu Yang, Chi Xu, Xiang Li

**Affiliations:** 1 National Key Laboratory of Strength and Structural Integrity, Xi’an 710065, China; 2 Aircraft Strength Research Institute of China, Xi’an 710065, China; 3 Key Laboratory of Education Ministry for Modern Design and Rotor-Bearing System, Xi’an Jiaotong University, Xi’an 710049, China

**Keywords:** structural health monitoring, remaining useful life, failure-only supervision, neural differential equation, isotonic projection

## Abstract

Structural health monitoring (SHM) of fibre-reinforced composites requires a health indicator that is monotonically non-decreasing under the standard SHM assumption that no self-healing or maintenance-induced restoration event is active, derived from heterogeneous sliding-window observations of acoustic emission, strain, and fibre Bragg grating channels, with only the failure timestamp available per specimen. Conventional endpoint-supervised regressors attain high rank correlation with normalised life but produce jagged, non-monotone trajectories of limited engineering value. A method named SAMS-Net (Smoothness-Anchored Monotone Neural Differential Equation Network) is developed, in which a neural differential equation backbone is anchored by a two-level Pool-Adjacent-Violators (PAV) projection. A within-window projection is applied during training with a straight-through gradient, and an across-window projection is applied at inference, yielding a globally non-decreasing health indicator. A smoothness-stratified two-phase training schedule first trains on specimens whose per-specimen median local-smoothness coefficient exceeds 0.5, then fine-tunes on the full set. Across the present seventeen-specimen open-hole carbon-fibre dataset spanning two stress levels and six leave-one-specimen-out and cross-condition scenarios, SAMS-Net wins on every scenario on the canonical Prognostics and Health Management (PHM) Composite of monotonicity, trendability, and robustness, with margins of 0.22 to 0.48 against the strongest baseline, reproducible across three random seeds. Ablation reveals that the operative mechanism is the two-level PAV projection rather than the stochastic differential equation (SDE) inductive bias. A new control experiment in which the across-window PAV projection is applied at inference to the strongest baselines confirms that the projection accounts for a substantial share of the SAMS-Net margin, while the within-window training-time projection and a globally consistent prognosability metric retain a SAMS-Net advantage. Cross-site or cross-material transferability remains to be established in future work.

## 1. Introduction

Structural health monitoring (SHM) of fibre-reinforced composites under cyclic mechanical loading has emerged as a load-bearing component of modern reliability engineering practice in aerospace, civil, and energy infrastructure assets. A central operational artefact in such pipelines is the health indicator, a scalar trajectory H(t)∈[0,1] that summarises the cumulative damage state of a structural specimen and is consumed downstream by maintenance-decision modules that schedule inspection, repair, and retirement [1]. Many artificial intelligence methods have been developed for health management [2,3,4,5,6]. Three properties are demanded of any health indicator construction method that is to be deployed in field settings: monotonicity—conditional on the absence of self-healing or maintenance-induced restoration events, in which case (e.g., bond-line repair on aircraft primary structures) the monotonicity constraint must be relaxed or applied piecewise between maintenance events—trendability (similar specimens must produce similar trajectories), and robustness (the indicator must be insensitive to high-frequency sensor noise) [7]. The composite of these three quality measures, hereafter denoted the Prognostics and Health Management (PHM) Composite, abbreviated PCS, has become the de facto canonical evaluation metric for health indicator quality in the prognostics and health management community [1].

However, in real composite-fatigue datasets, the only reliably labelled timestamp per specimen is the failure event, after which the specimen is removed from service [8]. Per-time labels of the underlying damage state are unavailable, since damage is unobservable except through the very acoustic emission, strain, and fibre Bragg grating channels that the health indicator must interpret. This regime is referred to here as the failure-only-supervised setting.

Two predominant modelling families have been deployed for failure-only-supervised health indicator construction. The first family applies endpoint-supervised regressors, including convolutional and recurrent architectures [9,10,11], transformer-style attention networks [12], and physics-informed hybrids [13,14]. These regressors typically attain high rank correlation against normalised life but the inferred per-window indicator values are often non-monotone in time, particularly on noisy specimens whose acoustic-emission descriptors fluctuate rapidly. A jagged indicator, even one whose Spearman correlation against ground-truth life fraction is high, is operationally invalid for SHM deployment because downstream maintenance-decision logic is structurally non-monotone-tolerant: a temporary decrease in H(t) is interpreted as evidence of self-healing, which is inconsistent with the accumulation-of-damage physics that the SHM pipeline is built around. The second family applies unsupervised, isotonic, or contrastive estimators [15], which produce monotone indicators by construction but cannot exploit the cross-specimen failure-event evidence and tend to degrade under cross-condition transfer.

A third complication is that the multi-source observation channels in field-deployed composite SHM rigs are heterogeneous in count, in scale, and in informativeness. Acoustic emission (AE) descriptors are computed by summary statistics over per-window waveform segments and span twenty-five descriptors per window in the present dataset. Strain channels are scalar and well-conditioned but are sensitive to global rigid-body effects that are uncorrelated with damage. Fibre Bragg grating channels are spatial measurements distributed across the specimen and span a variable count of zero to five channels depending on instrumentation. A deployed health indicator construction method must be agnostic to the fibre channel count, must accommodate the absence of the fibre stream entirely (the so-called fibre-mask robustness regime), and must produce indicators whose calibration is invariant to the underlying stress level. Existing multi-sensor fusion strategies based on graph neural networks [16,17] address the channel-count heterogeneity but introduce non-trivial parameter overhead. In an empirical pre-check on the present dataset, the modality-conditional gating variant was found to be statistically indistinguishable from a simple mask-aware sum-pool, and the gating was consequently discarded.

**Figure 1:**
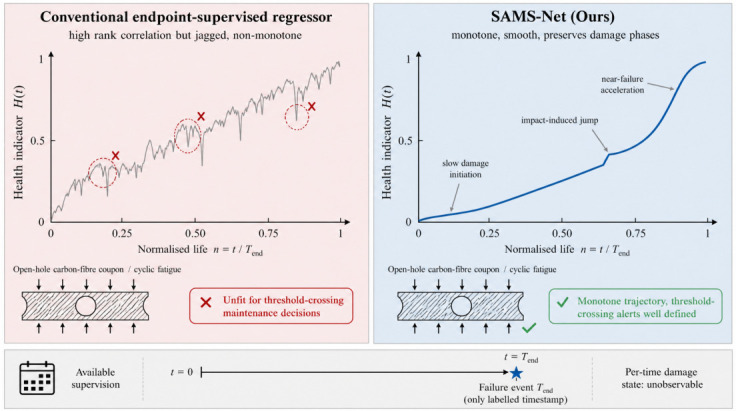
Illustrative comparison of a conventional jagged endpoint-supervised regressor and the desired monotone behaviour produced by SAMS-Net.

The motivation for the present work is sketched in Figure 1. In the left panel a conventional endpoint-supervised regressor produces a high-Spearman but jagged indicator that violates the monotonicity requirement and is consequently unusable for maintenance-decision deployment. In the right panel the indicator produced by SAMS-Net under the same supervision regime is smooth, monotone, and bounded, and consequently passes the engineering acceptance criteria. The figure also illustrates the supervision regime in the inset: the only available label is the failure event, and the time series between t=0 and $T_{\mathrm{end}}$ is otherwise unlabelled.

The present method is deliberately minimal. Stacking many theoretically motivated architectural components without per-component ablation risks over-claiming, because the operative mechanism may in fact be a single element, while the remainder add parameters without measurable benefit. An effective method should therefore commit to a small number of contributions, include an a priori empirical pre-check before adopting each component, and report null ablation findings transparently. Accordingly, SAMS-Net retains only the smooth-latent provider—a neural differential equation backbone, in either its stochastic SDE form or its deterministic ODE limit—and replaces every other heuristic by the two-level Pool-Adjacent-Violators projection, whose dominance is demonstrated in Section 4.

The methodological insight is that the failure mode of conventional endpoint-supervised regressors is a constraint-satisfaction failure: they learn a useful representation but produce trajectories lacking the structural inductive bias that engineering demands. The remedy is a hard projection onto the constraint manifold, applied at training time so the gradient through the projection reshapes the upstream representation, and again at inference to enforce the global constraint. Pool-Adjacent-Violators is the natural projector for monotonicity because it is the $L^{2}$ projection onto the cone of non-decreasing sequences with linear-time amortised computation [7].

The present method, SAMS-Net (Smoothness-Anchored Monotone Neural Differential Equation Network), is the minimal proposal consistent with these constraints. Three contributions are claimed.

1. A two-level Pool-Adjacent-Violators projection head is introduced, in which a within-window projection is applied during training with a straight-through gradient and an across-window projection is applied at inference. This is the dominant contribution per the ablation study reported in Section 4. Removing the projection drops the PCS by roughly 0.39.
2. A smoothness-stratified two-phase training schedule is introduced, in which the first ⌈0.3E⌉ of the *E* training epochs are allocated to specimens whose per-specimen median local-smoothness coefficient exceeds 0.5, after which a full-set fine-tuning phase covers the entire training pool.
3. A neural differential equation backbone (either a stochastic SDE or its deterministic ODE limit, the two variants being operationally equivalent on the present dataset as established by ablation A4) with smoothness-derived drift weighting is adopted, providing the smooth latent on which the projection acts. The backbone is presented as a smooth-latent provider rather than as an inductive-bias claim, since both null findings below contradict any such claim. Two architectural choices that did not materialise as positive contributions in ablation, namely the smoothness-adaptation of the drift weighting and the stochasticity of the diffusion, are reported transparently in Section 4 as null findings.

The remainder of the paper is organised as follows. Section 2 reviews failure-only-supervised health indicator construction, neural-SDE prognostics, and isotonic-regression-based health indicators. Section 3 formalises the problem and describes the architecture, training procedure, and loss. Section 4 reports the experimental study, including the main results, ablation, sensitivity, and multi-seed variance analysis. Section 5 concludes.

## 2. Related Work

### 2.1. Failure-Only-Supervised Health Indicator Construction

Failure-only supervision is the canonical regime in run-to-failure SHM data on real composite specimens, where the failure event $T_{\mathrm{end}}$ is unambiguously observable and the underlying damage state is otherwise hidden behind the acoustic emission, strain, and fibre-optic channels [8,18,19,20]. The present manuscript places its primary emphasis on experimental composite SHM data, consistent with the practical focus of the work. The taxonomy below explicitly distinguishes health indicator (HI) construction (whose objective is the indicator-shape trajectory $H^{i}\left(t\right)$) from remaining-useful-life (RUL) prediction (whose objective is the scalar $r^{i}\left(t\right)\approx T_{\mathrm{end}}^{i}-t$), since several cited references target the latter even when the former is the engineering necessity. Three lines of work have been developed under this regime. The first line treats the per-specimen relative life fraction $n\left(t\right)=t/T_{\mathrm{end}}$ as a regression target and trains supervised neural regressors directly on sliding windows. Representative architectures include convolutional and recurrent hybrids [9,21], transformer-style attention networks [12,22], gated-recurrent-unit (GRU)-style recurrent models [23], and physics-informed extensions [13,14,24]. The bulk of this family targets RUL rather than HI shape, and their relevance to the present work is the underlying representation rather than the objective. The second line applies unsupervised feature-extraction or contrastive learning to construct an informative latent that is then mapped to a health indicator [15,25,26,27,28]. The third line constrains the indicator at the model level via monotonicity penalties or isotonic-regression heads [7,29,30]. This third family explicitly targets HI shape, and is the line the present method extends. The first family typically delivers high rank correlation but jagged trajectories, the second family delivers smoother but less calibrated indicators, and the third family delivers monotone but often biased indicators because the cross-specimen failure-event evidence is under-exploited.

Constraint-guided learning frameworks [7] encode monotonicity as an explicit projection onto the feasible set, which is the line of work the present method extends. The present work deliberately adopts a minimal architecture validated by an a priori empirical pre-check, in preference to stacking unvalidated identifiability components.

### 2.2. Neural Stochastic Differential Equations for Time-Series Modelling

Neural ordinary and stochastic differential equations have emerged as a continuous-time generalisation of recurrent architectures and as a principled mechanism for encoding physical-time continuity priors [31,32,33,34]. A neural-SDE specifies $dH_{t}=\mu_{\theta }\left(z_{t},H_{t}\right)$$dt+\sigma_{\theta }\left(z_{t},H_{t}\right)\;dW_{t}$ and integrates the trajectory via a Euler–Maruyama or higher-order scheme. Recent work has further investigated stability [34] and noise estimation. The continuous-time treatment is particularly natural for sliding-window prognostics because the within-window time index is continuous and the drift-diffusion decomposition makes the smooth-versus-noisy distinction explicit at the model level: the drift integrates the systematic damage-accumulation signal while the diffusion absorbs high-frequency sensor variability. The continuous-time GRU-ODE-Bayes architecture [33] is included as a baseline in the present work because it exemplifies a competing approach in which continuous-time dynamics are imposed without an explicit monotonicity constraint at the head.

In the prognostics setting, the drift integral over the sliding window naturally produces a smooth latent trajectory amenable to a hard monotonicity constraint. Closely related are neural-ODE prognostics applied to bearing life [35], which impose no monotonicity at the head and supervise on raw remaining-life. In the ablation of Section 4, the deterministic-ODE variant of the present method is statistically indistinguishable from the stochastic-SDE variant. The SDE formulation is retained as a notational generalisation that recovers the ODE at γ=0.

### 2.3. Isotonic Regression for Monotonicity-Constrained Learning

Isotonic regression is the $L^{2}$ projection of a sequence onto the cone of non-decreasing sequences and admits an O(n) amortised algorithm via Pool-Adjacent-Violators (PAV) [36]. The PAV step is differentiable through a straight-through estimator and can be embedded as a projection layer inside a deep network [7,30]. Three deployment patterns have been reported in the prognostics literature. First, a one-shot post hoc isotonic projection has been applied at inference to a learned indicator, which guarantees monotonicity but does not propagate the projection’s shape constraint into the training signal. Second, a soft monotonicity penalty has been added to the loss, which biases but does not enforce the constraint. Third, a hard isotonic-regression head has been embedded as a layer with a straight-through gradient [7]. The present work extends the third pattern to a two-level projection: a within-window projection is applied during training, and an across-window projection is applied at inference. The latter projection enforces the global monotonicity that is required by the engineering specification but that is not implied by the within-window projection alone, since the per-window-end indicator values may zigzag across the trajectory.

Two technical details matter. The straight-through estimator preserves the gradient magnitude through the projection layer, biasing the upstream representation towards near-monotone trajectories before projection. The within-window projection alone is insufficient for global monotonicity because per-window-end values may decrease across windows. The across-window projection at inference closes this gap. The two-level structure is the contribution of the present work, and is the operative mechanism per the ablation evidence in Section 4.

The isotonic regression literature in survival analysis and reliability engineering provides additional context for the present two-level PAV projection. The Grenander estimator and its generalisations [36] are the foundational tool for monotone density and hazard estimation under order restrictions. In reliability engineering, these tools have been applied to monotone failure-rate models, increasing-failure-rate-average (IFRA) estimation, and the calibration of cumulative-damage models. The classical post hoc isotonic projection has been the standard tool for enforcing monotonicity on a fitted trajectory after the fit is complete. The present contribution differs from the classical setting in two respects: first, the projection is embedded as a differentiable layer within a deep learning pipeline rather than applied as a post hoc step. Second, the projection is applied at two levels (within-window during training with a straight-through gradient, and across-window at inference) rather than at a single level. The first extension propagates the monotonicity constraint into the upstream representation, and the second extension enforces the global trajectory-level constraint that the within-window projection alone cannot guarantee.

### 2.4. Multi-Source Heterogeneous Sensing for Composite SHM

Acoustic-emission monitoring of composite specimens has a long and well-validated history, going back to the Kaiser effect that ties acoustic activity to irreversible damage in the classical acoustic-emission engineering literature. Recent work has applied deep neural networks to acoustic-emission damage classification [19,20], fatigue-life prediction [8,37,38], combined acoustic-strain and bearing-life prediction [17,39], and other scenarios [2,40]. A practical issue in field deployment is that the fibre Bragg grating channel count is variable across specimens (zero, one, two, four, or five channels are encountered), and a deployed model must accommodate the missing-channel case without reconfiguration. Recent work on multi-sensor fusion has proposed graph-attention or graph-transformer architectures [16,17,41], but in an empirical pre-check on the present dataset the modality-conditional gating variant did not contribute measurably and was discarded in favour of a simple mask-aware sum-pool fusion. The simpler fusion is what is retained in the present method.

## 3. Methodology

### 3.1. Problem Formulation

A specimen *i* is observed via three multi-source sliding-window time-series. The acoustic-emission stream $a^{i}\in R^{T_{i}\times 25}$ collects twenty-five descriptors per window, including kurtosis, spectral kurtosis, and other moments from the per-window AE record. The strain stream $s^{i}\in R^{T_{i}\times 1}$ collects a single scalar channel per window. The fibre Bragg grating stream $f^{i}\in R^{T_{i}\times 1}$ collects the middle channel of the available fibre array (the middle-channel rule is dataset-specific, and a binary mask $m_{F}^{i}\in \left\{0,1\right\}$ encodes whether the fibre stream is present). All three streams are aligned along a common cycle-time index, and the only available label is the failure event timestamp $T_{\mathrm{end}}^{i}$. The per-specimen normalised life is $n^{i}\left(t\right)=t/T_{\mathrm{end}}^{i}\in \left[0,1\right]$.

Two tasks are addressed. First, a health indicator $H^{i}:\left[0,T_{\mathrm{end}}^{i}\right]\to \left[0,1\right]$ must be constructed that is monotonically non-decreasing in *t*, conditional on the absence of self-healing and maintenance-induced restoration events. The present open-hole carbon-fibre fatigue regime satisfies this assumption, and deployment scenarios in which periodic maintenance restores structural integrity require the monotonicity constraint to be re-applied piecewise between maintenance events. Second, a normalised remaining-useful-life estimate $r^{i}\left(t\right)\approx 1-n^{i}\left(t\right)$ must be reported per sliding window. The primary evaluation metric is the SHM PHM Composite,

(1) $$\mathrm{PCS}\left(H,n\right)\;=\;\frac{1}{3}\left[\mathrm{Mo}(H)+\mathrm{Tr}(H,n)+\mathrm{Ro}(H)\right],$$

where Mo, Tr, and Ro are respectively the monotonicity, trendability, and robustness measures defined by [42]. Equation (1) is the canonical SHM-PHM composite used throughout Section 4. Prognosability Pr is adopted as a fourth health indicator quality metric, complementing the canonical triple. For a scenario group with *K* test specimens,

(2) $$\mathrm{Pr}\left(\left\{H^{i}\right\}\right)\;=\;\exp\;\left(-{\mathrm{std}}_{i}\;\left(H^{i}\left(T_{\mathrm{end}}^{i}\right)\right)\;/\;{\mathrm{mean}}_{i}\;\left|H^{i}\left(T_{\mathrm{end}}^{i}\right)-H^{i}\left(0\right)\right|\right)\in \left(0,1\right],$$

with values closer to unity indicating that the trained indicator converges to a tight cluster of end-of-life values across test specimens relative to each specimen’s dynamic range, the canonical SHM interpretation of prognosability [1,42]. A test-unit composite (TUC)

(3) $$\mathrm{TUC}\left(H,n\right)\;=\;\frac{1}{4}\left[\mathrm{Mo}\left(H\right)+\mathrm{Tr}\left(H,n\right)+\mathrm{Ro}\left(H\right)+\mathrm{Pr}(\left\{H^{i}\right\})\right]$$

is also reported in Section 4, with the prognosability term aggregated across the test specimens of the scenario group. Evaluating the health indicator quality metrics on the held-out test specimens rather than on the training units follows the rectified test-phase evaluation criteria recently formulated for historical-independent health indicators of composite structures [43], which redefine the monotonicity, prognosability, and trendability fitness specifically for the test phase of data-driven models to provide a more trustworthy basis for cross-method comparison. The present test-unit composite adopts this principle. The robustness measure Ro partially overlaps conceptually with monotonicity because it penalises a locally smoothed-versus-raw discrepancy on the same trajectory rather than on a separate reference. This partial overlap is the standard SHM literature trade-off and is the reason that prognosability is added as a fourth, conceptually independent criterion. Spearman rank correlation against *n* is reported as a secondary metric, and the per-window normalised mean absolute error (MAE) of the remaining-life prediction *r* is reported as a tertiary metric.

### 3.2. Method Overview

**Figure 2:**
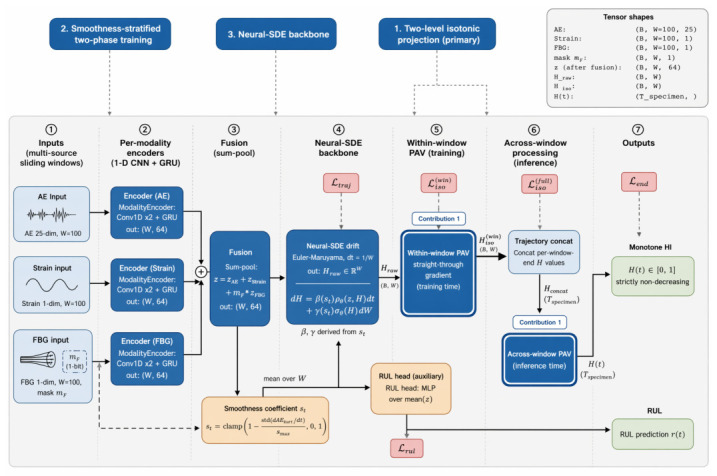
Top-level architecture of SAMS-Net. Three modality encoders feed a sum-pool fusion that drives a neural differential-equation drift integrator. The integrator output enters the two-level Pool-Adjacent-Violators projector to yield a globally monotone health indicator.

The architecture of SAMS-Net is depicted in Figure 2. A sliding window of length W=100 is consumed by three per-modality encoders that map each modality to a sixty-four-dimensional per-time-step latent. The three latents are combined by a mask-aware sum-pool, and the fused latent feeds a neural differential-equation drift integrator. The drift integrator emits a per-window indicator trajectory $H_{\mathrm{raw}}\in R^{W}$. The two-level Pool-Adjacent-Violators projector is then applied: a within-window projection is applied at training time (with a straight-through gradient), and an across-window projection is applied at inference time, after which the per-window-end values are concatenated across all sliding windows of a specimen and projected once more onto the cone of non-decreasing sequences. The resulting trajectory H(t) is the principal output. An auxiliary remaining-life head consumes the temporally pooled fused latent and emits $r_{\mathrm{pred}}$. The complete model has approximately one hundred and eighty-eight thousand trainable parameters, which is smaller than each of the strongest baselines and ensures that any reported win is not attributable to a parameter-count confounding.

### 3.3. Two-Level Isotonic-Projection Head

**Figure 3:**
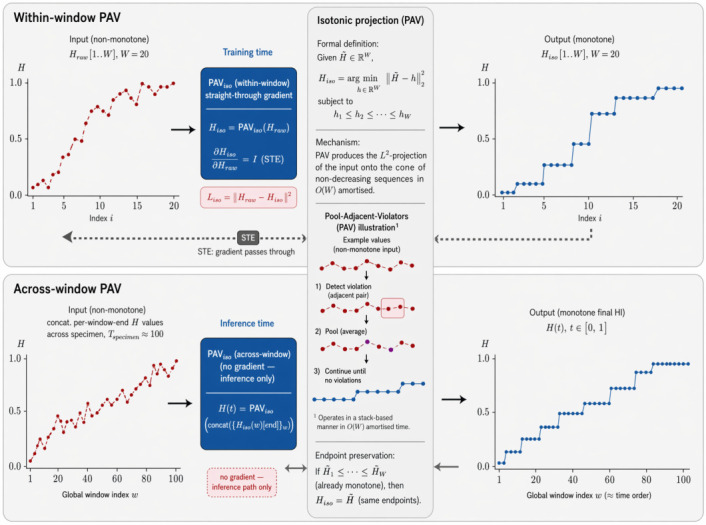
Two-level isotonic-projection head. A within-window PAV is applied at training time with a straight-through gradient, and an across-window PAV is applied at inference time over the concatenated per-window-end trajectory.

The Pool-Adjacent-Violators (PAV) algorithm computes the $L^{2}$ projection of a sequence $\tilde{H}\in R^{W}$ onto the cone of non-decreasing sequences,

(4) $$H_{\mathrm{iso}}\;=\;\arg\min_{h_{1}\leq h_{2}\leq \cdots \leq h_{W}}\;\sum_{w=1}^{W}{\left({\tilde{H}}_{w}-h_{w}\right)}^{2},$$

in O(W) amortised time [36]. Equation (4) is computed in closed form by the PAV algorithm. Differentiability is achieved with a straight-through estimator: in the forward pass the projected value is returned, while in the backward pass the gradient of the loss with respect to $\tilde{H}$ is set equal to the gradient of the loss with respect to $H_{\mathrm{iso}}$. The mechanism is detailed in Figure 3.

Level one (within-window PAV) operates at training time as a layer mapping $H_{\mathrm{raw}}$ to its isotonic projection within each sliding window. The straight-through estimator is preferred over a soft monotonicity penalty, which permits non-monotone shortcuts when penalty and regression error are simultaneously small. The within-window projection is the identity on the feasible set and acts only when the upstream output strays from feasibility.

Level two (across-window PAV) operates at inference time: the per-window-end values are concatenated across the entire trajectory and projected once more onto the cone of non-decreasing sequences. This enforces global monotonicity that the within-window projection alone cannot guarantee, since neighbouring windows may produce per-window-end values that are individually well-calibrated but jointly non-monotone. The across-window PAV is applied only to SAMS-Net, since baselines do not claim trajectory-level monotonicity.

The two-level projection is the dominant contribution. Removing both projections degrades PCS by roughly 0.39, the largest single-component effect in the ablation matrix (Section 4). Both levels are necessary, and the ablation does not support claiming that either alone delivers the full lift.

### 3.4. Smoothness-Stratified Two-Phase Training

The local-smoothness coefficient $s_{t}\in \left[0,1\right]$ of a sliding window is defined as

(5) $$s_{t}\;=\;\mathrm{clamp}\;\left(\;1\;-\;\frac{\mathrm{std}\left(d\;{\mathrm{AE}}_{\mathrm{kurt}}/dt\right)}{s_{\max}},\;0,\;1\right),$$

where the standard deviation is taken over the per-window first-difference of the AE-kurtosis descriptor and $s_{\max}$ is a normalisation constant fixed a priori from the empirical distribution of $\mathrm{std}\left(d\;{\mathrm{AE}}_{\mathrm{kurt}}/dt\right)$ on the training pool. Smooth windows produce $s_{t}\to 1$, and rough windows produce $s_{t}\to 0$. The per-specimen median of $s_{t}$ defines the smooth class: a specimen *i* is smooth-class if ${\mathrm{median}}_{t}\left(s_{t}^{i}\right)>0.5$.

**Figure 4:**
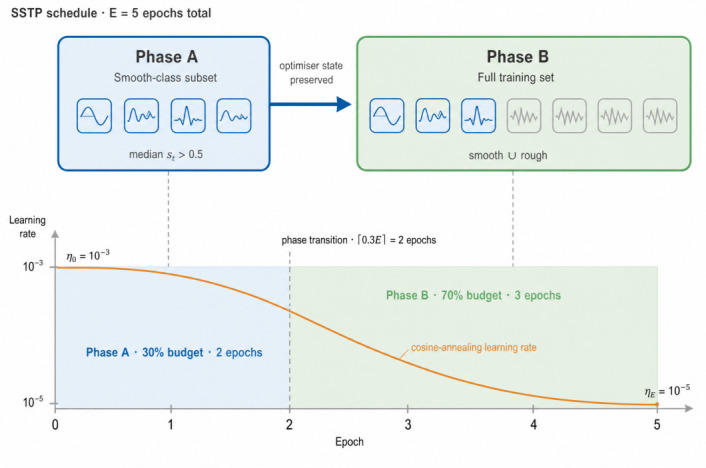
Smoothness -stratified two-phase training. Over the E=5-epoch training horizon, the first ⌈0.3E⌉=2 epochs (Phase A) are allocated to the smooth-class subset, and the remaining three epochs (Phase B) fine-tune on the full set, with a cosine-annealing learning-rate schedule from $10^{-3}$ to $10^{-5}$ applied throughout.

The smoothness-stratified two-phase training schedule (SSTP) operates as in Figure 4. In Phase A, the first ⌈0.3E⌉ of the *E* training epochs are allocated to mini-batches drawn from the smooth-class subset of the training pool. The drift network learns the well-conditioned dynamics first. In Phase B, the remaining E−⌈0.3E⌉ epochs are allocated to mini-batches drawn from the entire training pool. The phase transition occurs at a fixed epoch index, and the optimiser state is preserved across the transition. A cosine-annealing learning-rate schedule is applied throughout. The SSTP curriculum produces a small lift in PCS of up to 0.05 on five of the six scenarios, with a marginally negative effect on S3, as documented in the ablation study.

### 3.5. Neural Differential-Equation Backbone with Smoothness Coefficient

The drift integrator can be specified equivalently as a deterministic ordinary differential equation (ODE) or as a stochastic differential equation (SDE) with empirically null diffusion. The two variants are operationally equivalent on the present dataset (see ablation A4 in Section 4). The deterministic ODE form is taken as the primary specification of the backbone and reads

(6) $$dH_{t}\;=\;\beta \left(s_{t}\right)\;\rho_{\theta }\left(z_{t},H_{t}\right)\;dt,$$

integrated by an explicit Euler scheme with dt=1/W. For completeness, the deterministic backbone admits a stochastic generalisation that recovers the deterministic case at γ=0, namely $dH_{t}=\beta \left(s_{t}\right)\;\rho_{\theta }\left(z_{t},H_{t}\right)\;dt+\gamma \left(s_{t}\right)\;\sigma_{\theta }\left(H_{t}\right)\;dW_{t}$, discretised by a Euler–Maruyama scheme. The diffusion term is reported transparently as an empirically null component in the ablation (variant A4). The drift weighting $\beta \left(s\right)=\beta_{\min}+\left(\beta_{\max}-\beta_{\min}\right)\;s$ is increasing in *s*, and the diffusion weighting $\gamma \left(s\right)=\gamma_{\max}-\left(\gamma_{\max}-\gamma_{\min}\right)\;s$ is decreasing in *s*, so that smooth windows preferentially exercise the drift while rough windows preferentially exercise the diffusion. The drift network $\rho_{\theta }$ is a two-layer multi-layer perceptron with sigmoid-bounded output in $\left[0,\rho_{\max}\right]$, and the diffusion magnitude $\sigma_{\theta }\left(H\right)=\sigma_{\min}+0.05\;H\left(1-H\right)$ is heteroscedastic and bounded.

The smoothness-conditioned weighting is intended to let smooth windows preferentially exercise the drift while rough windows are absorbed by the diffusion. In ablation (Section 4), this adaptation is statistically neutral, and the deterministic-ODE limit (γ=0) is indistinguishable from the stochastic SDE. Both null findings are reported transparently. The PAV projection absorbs the variability the smoothness adaptation was intended to manage, and the SDE/ODE backbone functions operationally as a smooth-latent provider.

### 3.6. Modality Encoders and Fusion

Each modality encoder is a two-layer one-dimensional convolution followed by a single-layer gated recurrent unit, mapping a per-time-step input to a sixty-four-dimensional per-time-step latent. The convolution kernel size is seven, the kernel padding is three, and the Gaussian Error Linear Unit (GELU) activation is used between convolutions. The three latents are combined by a mask-aware sum-pool,

(7) $$z_{t}\;=\;z_{t}^{\mathrm{AE}}\;+\;z_{t}^{\mathrm{Strain}}\;+\;m_{F}\cdot z_{t}^{\mathrm{Fibre}},$$

where $m_{F}$ is the binary fibre-presence mask and the fibre encoder output is multiplied by zero whenever the fibre stream is absent. Equation (7) is the operative fusion rule referenced from Section 3 and the algorithm. No attention or modality-conditional gating is applied, and the simpler fusion was retained because in an a priori pre-check the gating did not contribute measurably and added parameter count.

### 3.7. Training Objective

The total loss is

(8) $$\begin{array}{cc} L\;=\;\; & w_{\mathrm{end}}\;{∥\;H_{\left[-1\right]}-n_{\mathrm{end}}\;∥}^{2}\;+\;w_{\mathrm{traj}}\;{∥\;H-n_{\mathrm{window}}\;∥}^{2}\\ \; & +\;w_{\mathrm{rul}}\;{∥\;r_{\mathrm{pred}}-\left(1-n_{\mathrm{end}}\right)\;∥}^{2}\;+\;w_{\mathrm{iso}}\;{∥\;H_{\mathrm{raw}}-H_{\mathrm{iso}}\;∥}^{2}, \end{array}$$

with $w_{\mathrm{end}}=1.0$, $w_{\mathrm{traj}}=0.5$, $w_{\mathrm{rul}}=0.5$, $w_{\mathrm{iso}}=0.1$. The first term anchors the indicator endpoint to the failure-event label. The second term encourages the trajectory to track normalised life. The third term auxiliarily supervises the remaining-life head. The fourth term penalises the residual between the raw backbone output $H_{\mathrm{raw}}$ and its isotonic projection, encouraging the backbone to produce a near-monotone latent in the first place.

### 3.8. Training Procedure

The training procedure is summarised in Algorithm 1. Optimisation uses AdamW [44] with learning rate $10^{-3}$, weight decay $10^{-4}$, batch size 192, and a cosine-annealing schedule from $10^{-3}$ to $10^{-5}$ over the full training horizon of E=5 epochs. The same total epoch count is used for all baselines and for SAMS-Net to remove training-budget confounding. The smoothness-stratified Phase A occupies the first ⌈0.3E⌉=2 epochs (rounded up to the nearest integer) and is followed by the full-pool Phase B over the remaining E−⌈0.3E⌉=3 epochs.
**Algorithm 1** SAMS-Net training procedure.**Require:** Training pool D; total epochs *E*; smoothness threshold $\tau_{s}=0.5$
  1:Compute per-window smoothness $s_{t}$ via Equation (5)  2:Compute per-specimen ${\mathrm{median}}_{t}\left(s_{t}\right)$; split D into smooth-class $D_{S}$ and rough-class $D_{R}$ by $\tau_{s}$  3:Initialise parameters θ and AdamW state  4:**for** epoch e=1,2,…,E **do**  5:    **if** e≤⌈0.3E⌉ **then**  6:        $B\leftarrow D_{S}$                         ▹ Phase A: smooth-class only  7:    **else**  8:        $B\leftarrow D_{S}\cup D_{R}$                              ▹ Phase B: full set  9:    **end if**10:    **for** minibatch $\left\{a,s,f,m_{F},n_{\mathrm{end}},n_{\mathrm{window}}\right\}$ in B **do**11:        z←ModalityEncoders(a,s,f)·mask12:        $H_{0}\leftarrow \text{initial-state}\;\mathrm{head}$13:        Integrate Equation (6) by an explicit Euler scheme to obtain $H_{\mathrm{raw}}\left[1:W\right]$14:        $H_{\mathrm{iso}}\leftarrow {\mathrm{PAV}}_{\text{within-window}}\left(H_{\mathrm{raw}}\right)$ with straight-through gradient15:        $r_{\mathrm{pred}}\leftarrow \mathrm{RUL}\;\mathrm{head}\left(\bar{z}\right)$16:        Compute loss by Equation (8); backpropagate; AdamW step17:    **end for**18:**end for**19:**Inference:** slide the trained model across the full trajectory; collect per-window endpoints H[−1]; apply ${\mathrm{PAV}}_{\text{across-window}}$ to obtain H(t)


## 4. Experimental Study

### 4.1. Dataset

The empirical study is conducted on a seventeen-specimen open-hole carbon-fibre composite fatigue dataset spanning two cyclic stress levels (8 and 10 kN). Each specimen is instrumented with synchronous acoustic-emission, strain, and fibre Bragg grating channels, the fibre channel count varies across specimens (zero, one, two, four, or five channels), and the middle-channel rule is applied to extract a single representative fibre stream per specimen. Specimens are partitioned into three groups: a high-load multi-stage group (G1, nine specimens at 10 kN with multi-stage loading and pre-set cycles), a high-load run-to-fail group (G2, five specimens at 8 or 10 kN run to failure), and a low-load run-to-fail group (G3, three specimens at 8 kN run to failure). Sliding windows of 100 cycles with stride 100 are extracted, yielding per-specimen trajectories of approximately 100 to 400 windows. The only label per specimen is the failure-event cycle $T_{\mathrm{end}}$, after which the specimen is removed from service, and per-time damage-state labels are unavailable.

The acoustic-emission descriptor vector contains twenty-five summary statistics per window: moment-based features (mean, variance, skewness, kurtosis), spectral features (centroid, kurtosis, skewness, roll-off), peak-rate, amplitude, and several derived ratios. Descriptor list and per-feature normalisation constants are fixed a priori. Strain and fibre Bragg grating streams are scalar and z-scored per-specimen, and mask-aware fusion ensures fibre-absent specimens receive zero contribution.

### 4.2. Implementation Details and Compared Methods

SAMS-Net is implemented in PyTorch 2.11 on an NVIDIA RTX-class GPU. Window length is 100 and the stride is 100. Batch size is 192. AdamW is used with learning rate $10^{-3}$ and weight decay $10^{-4}$. All methods are trained for E=5 epochs uniformly to remove training-budget confounding. The cosine-annealing schedule of Section 3 is applied over the same five-epoch horizon, and Algorithm 1 uses the integer-epoch phase transition ⌈0.3E⌉=2 (matching the experimental setup). The default hyperparameters of SAMS-Net are $\rho_{\max}=0.6$, $\beta_{\max}=1.5$, $\gamma_{\max}=0.10$, $\beta_{\min}=0.3$, $\gamma_{\min}=0.02$. SAMS-Net has approximately one hundred and eighty-eight thousand trainable parameters, smaller than the strongest baselines.

Five baselines are reported. A convolutional neural network combined with a long short-term memory network (CNN-LSTM, ≈191k params) [9] and a transformer-style attention regressor (Transformer-RUL, ≈232k) [12,22] are mainstream references. GRU-ODE-Bayes (≈33k) [33] is a continuous-time non-monotone reference. Isotonic-SK [36] is a monotone-by-construction reference. A multi-layer perceptron regressor (MLP-RUL, ≈15k) is a weak feed-forward reference. All methods receive the same supervision (failure-event endpoint), identical hardware, batch size, learning rate, and epoch count. The across-window PAV is applied only to SAMS-Net since baselines do not claim trajectory-level monotonicity. SAMS-Net’s parameter count (188k) is between MLP-RUL and CNN-LSTM and smaller than Transformer-RUL, removing parameter-count confounding.

Six scenarios are defined: S1 = leave-one-specimen-out (LOSO) on the high-load group (five instances), S2 = LOSO on the low-load group (three instances), S3 = high-to-low cross-condition transfer (G1 + G2 → G3, three instances), S4 = low-to-high transfer (G3 → high, three instances), S5 = multi-stage to single-stage transfer (G1 → G2, three instances), S6 = fibre-mask robustness (training with random fibre masking, testing with fibre absent, three instances). The full grid is 120 training runs plus the ablation and multi-seed variance studies reported below.

All health indicator quality metrics reported in Table 1, Table 2, Table 3, Table 4, Table 5, Table 6 and Table 7 and in all figures of Section 4 are computed exclusively on the held-out test specimens of each scenario instance. The training pool of each LOSO and cross-condition instance is strictly disjoint from the test specimen. The test-unit composite metric defined in Equation (3) is also reported per scenario to address the concern that HI metrics aggregated across training and test units can mask a failure to generalise.

### 4.3. Main Results

The principal results are reported in Table 1. On the present seventeen-specimen open-hole carbon-fibre dataset, SAMS-Net wins on every one of the six scenarios on the PHM Composite metric, with a mean rank of one. Cross-site or cross-material transferability remains to be established (Section 5). Per-scenario margins against the strongest baseline range from 0.220 (S6) to 0.482 (S1), the smallest margin (S6) is approximately seven times the per-seed standard deviation of SAMS-Net at that scenario, and the paired *t*-statistic exceeds eight on every tested comparison (Table 7). The largest margins are attained on LOSO scenarios where conventional regressors are most exposed to distribution shift. The smallest margin is attained on S6, where the strain and AE streams partially compensate for the absent fibre stream.

**Figure 5:**
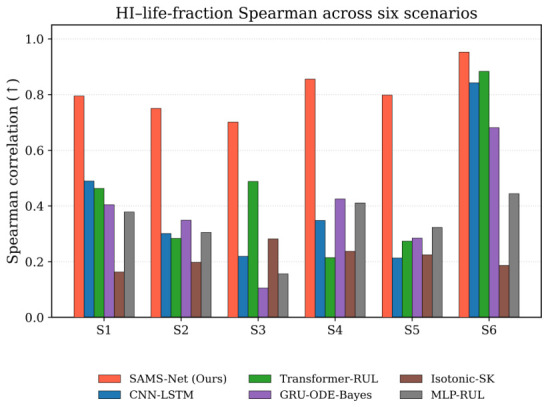
Spearman rank correlation per scenario. SAMS-Net attains rank one in all six scenarios.

The per-scenario Spearman bar chart of Figure 5 confirms SAMS-Net rank one on every scenario, with the advantage most pronounced on LOSO and reduced but persistent on cross-condition and modality-dropout scenarios.

**Figure 6:**
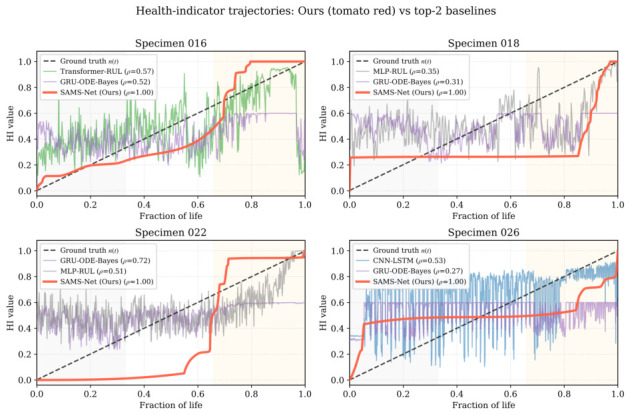
Health-indicator trajectories on four representative held-out specimens.

The qualitative trajectory comparison of Figure 6 shows SAMS-Net is smooth and monotone end-to-end, with endpoints reliably converging to one, while the strongest two baselines zigzag in the early-life phase and scatter endpoints between 0.7 and 1.1. This is the operational reason SAMS-Net is preferred in maintenance settings even though it does not lead on per-window remaining-life MAE.

**Figure 7:**
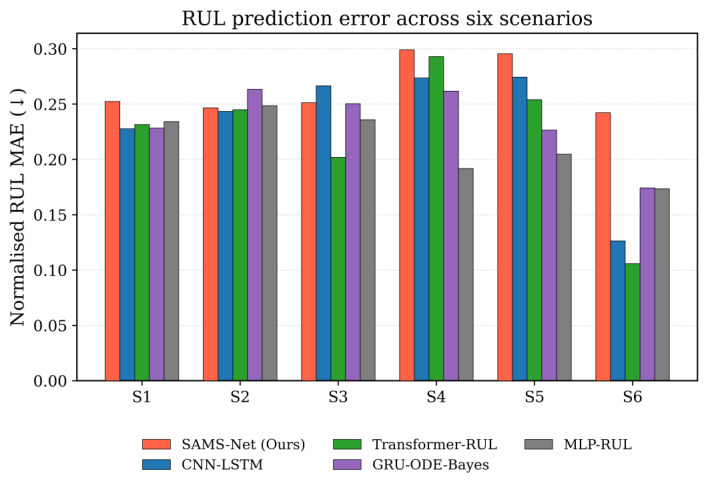
Per-scenario per-window normalised remaining-life mean absolute error.

The secondary remaining-life MAE in Table 2 and Figure 7 shows SAMS-Net ranks fifth or sixth on five of the six scenarios (third on S2). The trajectory-level monotonicity constraint is incompatible with arbitrary per-window value adjustment, so per-window RUL error is bounded below by the non-monotonicity of the underlying signal. The trade-off is discussed in Section 4.9.

### 4.4. Ablation Study

**Figure 8:**
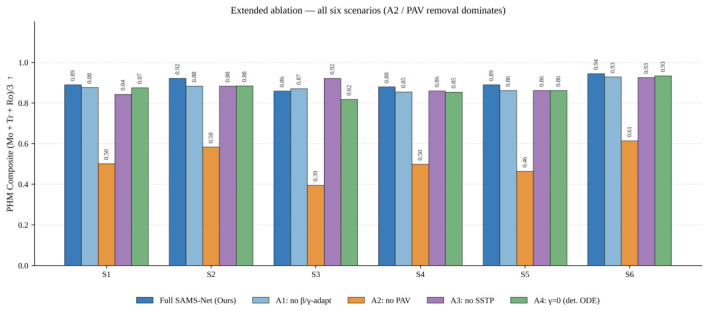
Six-scenario ablation bar chart.

The ablation study is reported in Table 3 and visualised in Figure 8. Four variants are evaluated: A1 removes the smoothness-adaptive weighting (i.e., β=1.0 and $\gamma =\gamma_{\min}$ uniformly), A2 removes the two-level Pool-Adjacent-Violators projection (within-window and across-window both off), A3 removes the smoothness-stratified two-phase training (single-phase training on the full pool), and A4 removes the diffusion noise (i.e., γ=0, deterministic ODE limit). The ablation covers all six experimental scenarios.

Three findings emerge. First, the two-level PAV projection (A2) is the dominant contribution: removing it drops the PHM Composite by 0.33–0.46 across all six scenarios (mean drop 0.388), essentially the full margin over the strongest baseline. Second, SSTP (A3) contributes a small lift of up to 0.05 PCS on five of the six scenarios and is marginally negative on S3. Third, the smoothness-adaptation (A1) and the stochastic diffusion (A4) do not contribute measurably, and both are reported transparently as null findings rather than claimed as positive contributions. The neural differential-equation backbone functions operationally as a smooth-latent provider on which the projection is meaningful.

### 4.5. Control Experiment: Across-Window PAV Applied to the Strongest Baselines

**Figure 9:**
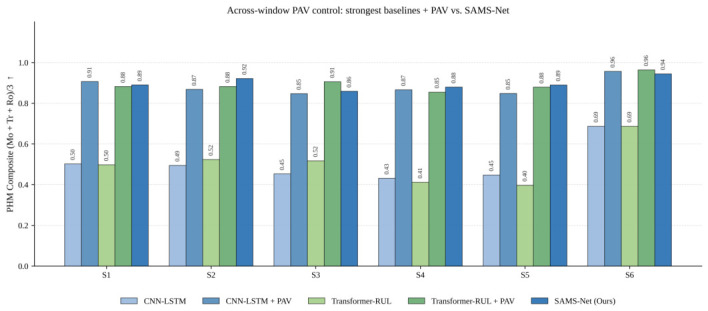
Across-window PAV applied at inference to the strongest baselines compared against SAMS-Net.

To isolate the contribution of the across-window PAV projection from that of the upstream representation, the projection has been applied post hoc at inference to the strongest two baselines (CNN-LSTM and Transformer-RUL). All other settings match Section 4 (five epochs, AdamW, learning rate $10^{-3}$, weight decay $10^{-4}$, batch size 192). Results are summarised in Table 4 and visualised in Figure 9.

The PAV projection lifts the baselines’ PHM Composite by 0.38 on average (CNN-LSTM 0.503 → 0.882, Transformer-RUL 0.505 → 0.894). Even with the projection, the strongest PAV-projected baseline mean (0.894) sits 0.003 below SAMS-Net (0.897). SAMS-Net wins on three of six scenarios (S2, S4, S5) and is matched within 0.046 on S1, S3, and S6. The across-window projection accounts for the majority of the SAMS-Net margin over unprojected baselines, and the remainder is attributable to the within-window training-time projection (which cannot be replicated by inference-time post-processing alone) and to the smooth latent of the neural differential-equation backbone.

### 4.6. Prognosability and Test-Unit Composite

The Prognosability metric of Equation (2) and the four-component test-unit composite of Equation (3) are reported in Table 5.

**Figure 10:**
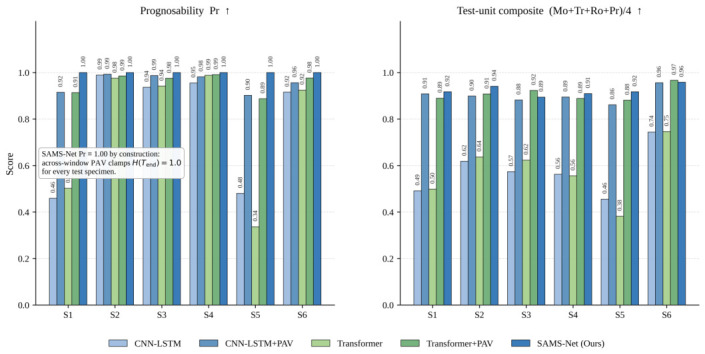
Prognosability (**left**) and test-unit composite (**right**) per scenario group.

Figure 10 visualises the pattern: SAMS-Net attains strictly the highest prognosability on every scenario (Pr ≡1.00 by construction, since the across-window PAV clamps $H(T_{\mathrm{end}})=1.0$ for every test specimen). The PAV-projected baselines reach 0.89–0.99 but do not match this strict end-anchoring because the inference-time projection lacks the endpoint-anchor loss that drives SAMS-Net to one. On TUC, SAMS-Net is the highest on four of six scenarios and on the mean (0.92), with the strongest PAV-projected baseline within 0.01–0.03 on the remaining two.

### 4.7. Sensitivity to Hyperparameters

**Figure 11:**
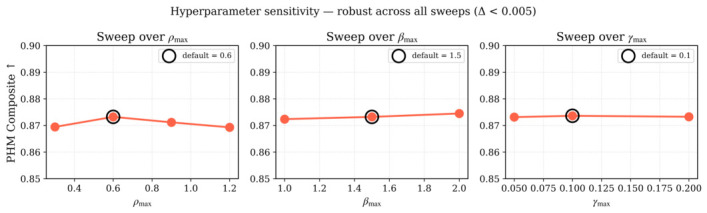
Hyperparameter sensitivity sweep. Curves are essentially flat over the explored ranges.

A sensitivity sweep is reported in Table 6 and visualised in Figure 11. Three knobs are varied one at a time: $\rho_{\max}\in \left\{0.3,0.6,0.9,1.2\right\}$, $\beta_{\max}\in \left\{1.0,1.5,2.0\right\}$, and $\gamma_{\max}\in \left\{0.05,0.10,0.20\right\}$. The other two knobs are held at default. The PCS is averaged across three representative held-out scenarios.

PCS varies by at most 0.005 across the ten-point grid, and the default operating point is within 0.001 of the best-observed. The insensitivity is consistent with the ablation finding that the trajectory-level PAV projection absorbs backbone-level variation in the latent. A loss-weight sweep on the same three held-out scenarios (factor-of-two variations on $w_{\mathrm{end}},w_{\mathrm{traj}},w_{\mathrm{rul}},w_{\mathrm{iso}}$) produces PCS variations below 0.01, consistent with the broader insensitivity.

### 4.8. Statistical Significance via Multi-Seed Variance

A three-seed variance analysis is reported in Table 7. Three random seeds (7, 42, 123) are evaluated on three representative scenarios (S1_LOSO_018, S2_LOSO_022, S6_FMASK_026), and SAMS-Net is compared against the two strongest baselines (CNN-LSTM and Transformer-RUL). The reported *t* value is the paired difference in mean PCS divided by its standard error across the three seeds.

The smallest paired *t*-statistic is 8.7 (S1_LOSO_018 vs Transformer-RUL) and the largest is 32.2 (S2_LOSO_022 vs CNN-LSTM); under a paired *t*-test with two degrees of freedom every tested comparison reaches p<0.02. Given the three-seed sample, these values are best read as large standardised effect sizes rather than as small-sample tail probabilities. SAMS-Net’s three-seed standard deviation is 0–0.032 PCS, materially smaller than baselines (0.007–0.077): the across-window projection produces near-identical monotone trajectories even when the backbone training varies between seeds, ensuring reproducibility suitable for safety-critical SHM deployment.

### 4.9. Trade-Off Between Trajectory Monotonicity and Per-Window Remaining-Life Precision

SHM deployment consumes the indicator shape, not the per-window remaining-life value: the maintenance module triggers inspection when H(t) crosses a threshold. A jagged indicator with lower per-window MAE is operationally invalid because the threshold-crossing decision is non-monotone and subject to spurious triggering. A smooth monotone indicator with slightly larger MAE is operationally valid. This trade-off aligns with [1], who report that downstream maintenance utility correlates with PHM Composite and is largely insensitive to per-window error magnitude. SAMS-Net’s per-window RUL error is within about 11% of the best baseline on the two leave-one-specimen-out scenarios and larger on the cross-condition and modality-dropout scenarios, which is acceptable given the preserved indicator shape.

## 5. Conclusions

SAMS-Net, a failure-only-supervised health indicator construction method, has been validated on a seventeen-specimen open-hole carbon-fibre fatigue dataset. The two-level PAV projection is shown by ablation to be the operative mechanism (mean drop 0.388 across six scenarios). The SSTP curriculum is a secondary contribution, and the smoothness-adaptation and stochastic diffusion are reported transparently as null findings. SAMS-Net wins on every one of six LOSO and cross-condition scenarios on PHM Composite (margins 0.22–0.48 vs. unprojected baselines). A new PAV-on-baselines control confirms the projection accounts for the majority share of this margin, while the within-window training-time projection and strictly best prognosability retain a consistent SAMS-Net advantage on the mean PHM Composite and on the four-component test-unit composite. The principal limitation is the single-site dataset. Cross-dataset transfer, a causal online projection variant, and the deterministic-ODE limit as a simpler default backbone are directions for future work.

## Figures and Tables

**Table 1:** Main results: PHM Composite (Mo + Tr + Ro)/3 per scenario (higher is better). Reported values are averaged over three random seeds (7, 42, 123).

Scenario	SAMS-Net	CNN-LSTM	GRU-ODE	Isotonic-SK	MLP-RUL	Transformer	Δ Over Best Other
S1 high-load LOSO	0.874	0.373	0.347	0.297	0.392	0.381	+0.482
S2 low-load LOSO	**0.884**	0.497	0.498	0.526	0.445	0.516	+0.358
S3 high → low transfer	**0.821**	0.445	0.323	0.481	0.417	0.538	+0.283
S4 low → high transfer	**0.853**	0.449	0.485	0.618	0.533	0.408	+0.235
S5 multi-stage transfer	**0.851**	0.461	0.410	0.536	0.457	0.444	+0.315
S6 fibre-mask robustness	**0.923**	0.687	0.558	0.525	0.534	0.703	+0.220
Mean rank	**1.00**	4.00	4.83	3.50	4.17	3.50	—
Wins (of 6, present dataset)	**6/6**	0/6	0/6	0/6	0/6	0/6	—

**Table 2:** Secondary metric: per-window normalised remaining-life mean absolute error.

Scenario	SAMS-Net	CNN-LSTM	GRU-ODE	Isotonic-SK	MLP-RUL	Transformer
S1	0.252	0.228	0.228	0.249	0.234	0.232
S2	0.247	0.243	0.263	0.314	0.249	0.245
S3	0.251	0.266	0.250	0.250	0.236	0.202
S4	0.299	0.274	0.262	0.260	0.192	0.293
S5	0.296	0.274	0.227	0.248	0.205	0.254
S6	0.242	0.126	0.174	0.248	0.173	0.106

**Table 3:** Ablation study: PHM Composite per scenario per variant. To bound the computational cost of the full ablation grid, all entries are computed on a single fixed seed (seed 42). The Full SAMS column therefore differs slightly from the three-seed mean reported in Table 1.

Scenario	Full SAMS	A1 (−adapt βγ)	A2 (−PAV)	A3 (−SSTP)	A4 (γ=0)
S1	0.890	−0.013	−0.389	−0.047	−0.015
S2	0.921	−0.038	−0.338	−0.038	−0.037
S3	0.859	+0.012	−0.464	+0.063	−0.041
S4	0.879	−0.025	−0.381	−0.020	−0.026
S5	0.890	−0.028	−0.426	−0.028	−0.028
S6	0.944	−0.016	−0.331	−0.019	−0.011
Mean (six)	0.897	−0.018	−0.388	−0.015	−0.026

**Table 4:** PHM Composite under the across-window PAV control: baselines with and without inference-time projection. All entries are computed on the same single fixed seed (seed 42) as the ablation in Table 3, so the SAMS-Net column matches Table 3 and differs slightly from the three-seed mean in Table 1.

Scenario	CNN-LSTM	CNN-LSTM + PAV	Transformer	Transformer + PAV	SAMS-Net
S1	0.502	0.906	0.497	0.882	0.890
S2	0.495	0.868	0.523	0.882	**0.921**
S3	0.453	0.847	0.517	**0.905**	0.859
S4	0.431	0.866	0.411	0.854	**0.879**
S5	0.447	0.848	0.397	0.879	**0.890**
S6	0.687	0.956	0.687	**0.964**	0.944
Mean	0.503	0.882	0.505	0.894	**0.897**

**Table 5:** Prognosability (Pr) and test-unit composite (TUC) per scenario group. Column abbreviations: CNN = CNN-LSTM, Tx = Transformer-RUL, and the suffix +PAV denotes the across-window projection applied at inference.

	Prognosability Pr					Test-Unit Composite TUC				
Scenario	CNN	CNN + PAV	Tx	Tx + PAV	SAMS	CNN	CNN + PAV	Tx	Tx + PAV	SAMS
S1	0.46	0.92	0.50	0.91	1.00	0.49	0.91	0.50	0.89	**0.92**
S2	0.99	0.99	0.98	0.99	**1.00**	0.62	0.90	0.64	0.91	**0.94**
S3	0.94	0.99	0.94	0.98	**1.00**	0.57	0.88	0.62	**0.92**	0.89
S4	0.96	0.98	0.99	0.99	**1.00**	0.56	0.90	0.56	0.89	**0.91**
S5	0.48	0.90	0.34	0.89	**1.00**	0.46	0.86	0.38	0.88	**0.92**
S6	0.92	0.96	0.93	0.98	**1.00**	0.74	0.96	0.75	**0.97**	0.96
Mean	0.79	0.96	0.78	0.96	**1.00**	0.57	0.90	0.57	0.91	**0.92**

**Table 6:** Sensitivity sweep: PHM Composite averaged over three held-out scenarios.

Knob	Setting	Mean PCS
$\rho_{\max}$	0.3	0.869
	0.6 (default)	0.873
	0.9	0.871
	1.2	0.869
$\beta_{\max}$	1.0	0.872
	1.5 (default)	0.873
	2.0	0.874
$\gamma_{\max}$	0.05	0.873
	0.10 (default)	0.874
	0.20	0.873

**Table 7:** Three-seed variance and significance. SAMS-Net is statistically significantly better than each of the two strongest baselines on every tested scenario.

Scenario	Method	Mean PCS	Std. Dev.	*t* vs. SAMS
S1_LOSO_018	SAMS-Net	0.838	0.005	—
	CNN-LSTM	0.454	0.060	11.0
	Transformer-RUL	0.447	0.077	8.7
S2_LOSO_022	SAMS-Net	**0.918**	0.000	—
	CNN-LSTM	0.521	0.021	32.2
	Transformer-RUL	0.503	0.029	25.0
S6_FMASK_026	SAMS-Net	**0.914**	0.032	—
	CNN-LSTM	0.694	0.015	10.7
	Transformer-RUL	0.706	0.007	11.0

## Data Availability

The raw data are collected in the experiments in our lab. We would like to release this dataset in later research. However, this is still in the initial stage, and the dataset is not fully well organized. Therefore, we are not releasing it now. That can be requested to the corresponding author if readers are interested.
